# Investigation of the Roles of Plasma Species Generated by Surface Dielectric Barrier Discharge

**DOI:** 10.1038/s41598-018-35166-0

**Published:** 2018-11-12

**Authors:** Kedar Pai, Chris Timmons, Kevin D. Roehm, Alvin Ngo, Sai Sankara Narayanan, Akhilesh Ramachandran, Jamey D. Jacob, Li Maria Ma, Sundararajan V. Madihally

**Affiliations:** 1 School of Mechanical and Aerospace Engineering, Oklahoma State University, Stillwater, OK 74078 USA; 2 Department of Entomology and Plant Pathology, Oklahoma State University, Stillwater, OK 74078 USA; 3 School of Chemical Engineering, Oklahoma State University, Stillwater, OK 74078 USA; 4 Oklahoma Animal Disease Diagnostics Laboratory, Oklahoma State University, Stillwater, OK 74078 USA

## Abstract

As an emerging sterilization technology, cold atmospheric plasma offers a dry, non-thermal, rapid process that is minimally damaging to a majority of substrates. However, the mechanisms by which plasma interacts with living cells are poorly understood and the plasma generation apparatuses are complex and resource-intensive. In this study, the roles of reactive oxygen species (ROS), nitric oxide (NO), and charged particles (ions) produced by surface dielectric barrier discharge (SDBD) plasma on prokaryotic (*Listeria monocytogenes* (Gram-positive)) and eukaryotic (human umbilical vein endothelial cells (HUVEC)) cellular function were evaluated. HUVEC and bacterial oxidative stress responses, the accumulation of nitrite in aqueous media, air ion density, and bacterial inactivation at various distances from SDBD actuators were measured. SDBD actuator designs were also varied in terms of electrode number and length to evaluate the cellular effects of plasma volume and power distribution. NO and ions were found to contribute minimally to the observed cellular effects, whereas ROS were found to cause rapid bacterial inactivation, induce eukaryotic and prokaryotic oxidative stress, and result in rapid oxidation of bovine muscle tissue. The results of this study underscore the dominance of ROS as the major plasma generated species responsible for cellular effects, with ions and RNS having a secondary, complimentary role.

## Introduction

Cold atmospheric plasma has been heavily investigated in recent years due to its many potential benefits in the field of healthcare, mainly for applications in disinfection and sterilization^1–5^, wound healing^6–8^, and cancer treatment^9–11^. Various cold plasma technologies have shown effectiveness against drug-resistant bacteria and are currently being reviewed for clinical applications^12–14^. Additionally, this technology has been extensively investigated in different forms for multiple food decontamination applications^15,16^. However, many of the current designs used to generate cold plasma rely on direct plasma exposure^14^, consist of complex apparatuses, and require an external gas flow for distribution of plasma-generated species to treatment sites^17–21^. As a result, there is a strong need for an effective, inexpensive, and versatile cold plasma generation technology with a simple design for broad applications in surface decontamination and sterilization.

As previously described^1,22^, surface dielectric barrier discharge (SDBD) is a novel method of non-thermal plasma generation that overcomes these drawbacks: it has low power requirements, greater treatment flexibility, and an increased effective treatment range. Since SDBD plasma generation is a semi-direct method of exposure to plasma species and does not require the substrate to complete the electric circuit, potential negative effects such as burning and tissue desiccation can be mitigated. SDBD exposure has shown a dose-based differential response in eukaryotic cells^23^ and lethal effects on prokaryotic cells^1,4,22,24^. It was observed that prokaryotic cells have a lower tolerance to plasma-generated species than eukaryotic cells^22,24^and therefore surface decontamination of eukaryotic tissues may be possible without adverse effects on the treated tissue. SDBD, being a surface treatment, may provide an alternative for precision surface treatments in hospitals, medical facilities, and dermatological applications.

Although investigated for many years for flow control applications^25–30^, the effects of different SDBD design parameters on the production and concentration of plasma-generated species has not been well defined. Different types and concentrations of plasma-generated species have direct effects on the responses of prokaryotic and eukaryotic cells to cold plasma treatments^21,24,31–33^. Thus, it is important to identify the SDBD design parameters that influence plasma-generated species production so that SDBD actuators can be optimized for specific decontamination and sterilization applications. Therefore, it was the goal of this study to evaluate the presence and concentration of ions, reactive nitrogen species (RNS), and reactive oxygen species (ROS) produced by SDBD and the influences of various design parameters on their production. More specifically, the effects of electrode length, electrode number, and the distance of treated surfaces from the electrodes were evaluated. Direct and indirect measurements were used to identify the cellular influence of these parameters and the importance of their biological effects was deduced. ROS and ions at close proximity to SDBD actuators were found to be the predominant plasma-produced species that influenced prokaryotic and eukaryotic cells. Furthermore, it was shown that optimization of SDBD actuator design can lower the power requirements and increase treatment effectiveness.

## Results and Discussion

### Correlation of Nitrite Production and Power Distribution

Nitric oxide (NO) is an important intracellular and intercellular signaling molecule involved in regulation of cardiovascular, nervous, and immunological function^34^. NO regulates vascular tone, endothelial permeability, smooth muscle cell proliferation, platelet aggregation, and other functions^34,35^. It is synthesized intracellularly during the conversion of L-arginine into L-citrulline in the presence of oxygen (O_2_), a reaction catalyzed by nitric oxide synthase (NOS). Nitrites (NO_2_^−^) are generated readily in aqueous solutions by oxidation of NO^36^ by O_3_ or O_2_^−^. For this reason, the production of extracellular NO following exposure to cold plasma was determined by measuring the accumulation of NO_2_^−^, the stable metabolite of NO secreted into the culture medium to provide evidence of reactive nitrogen species (RNS) produced by the plasma source^37,38^.

To evaluate the correlation of nitrite formation with power consumption, a two-electrode configuration was used, wherein the actuator consisted of two exposed and powered electrodes with a single encapsulated ground electrode on the opposite side of the dielectric. HUVEC media (without cells) in a 6-well plate was exposed to plasma at approximately 1 cm from the actuator for a treatment time of 2 minutes. The power per unit length was varied by altering the length of the electrodes. It was observed that the nitrite concentration increased with the increase in the power per unit length (Fig. 2a**)**. This result suggested a direct correlation of power with nitrite generation, and hence suggests that higher NO concentrations can be generated with a higher power input. These results agree with the findings of Pavlovich *et al*., who suggested a transition to higher NOx phase with increased power density^39^.

**Figure 1:**
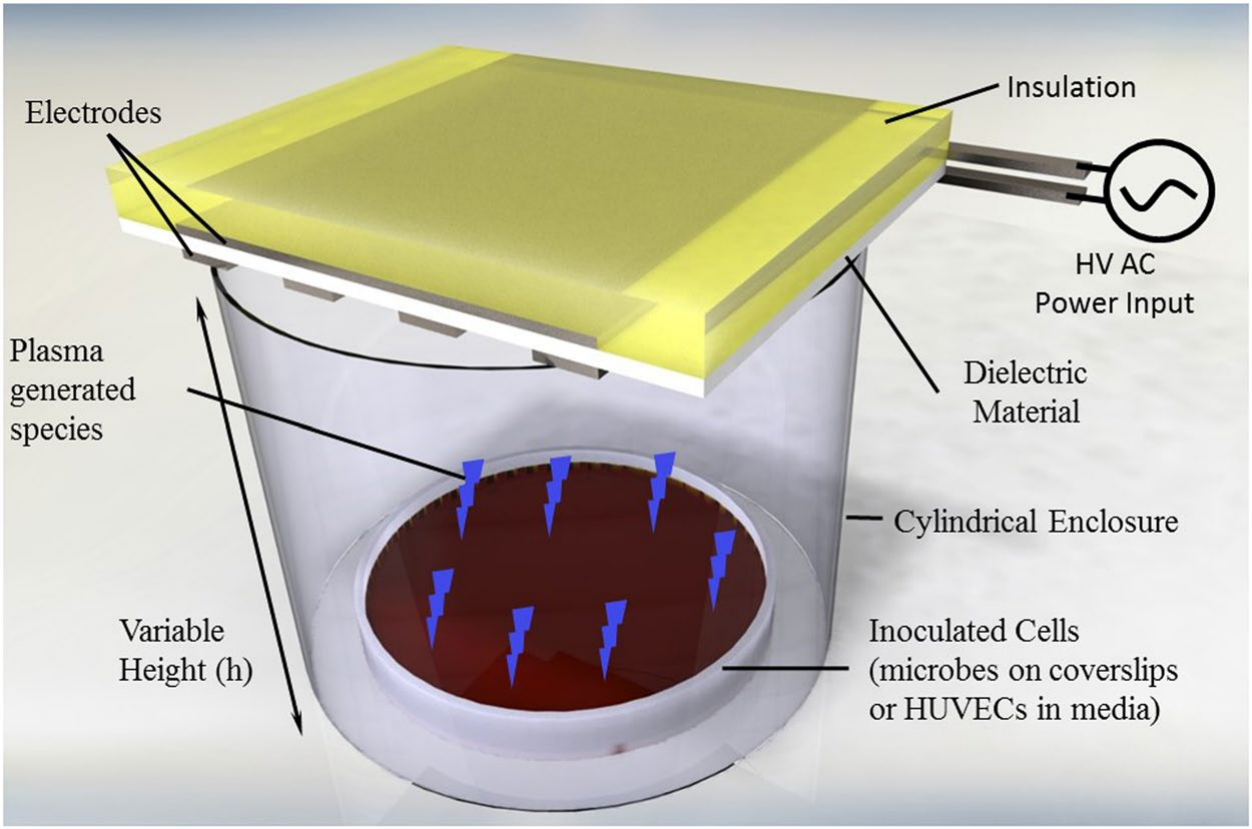
Schematic representation of surface dielectric barrier discharge (SDBD) cold plasma treatment apparatus used for prokaryotic and eukaryotic cells at various distances.

**Figure 2:**
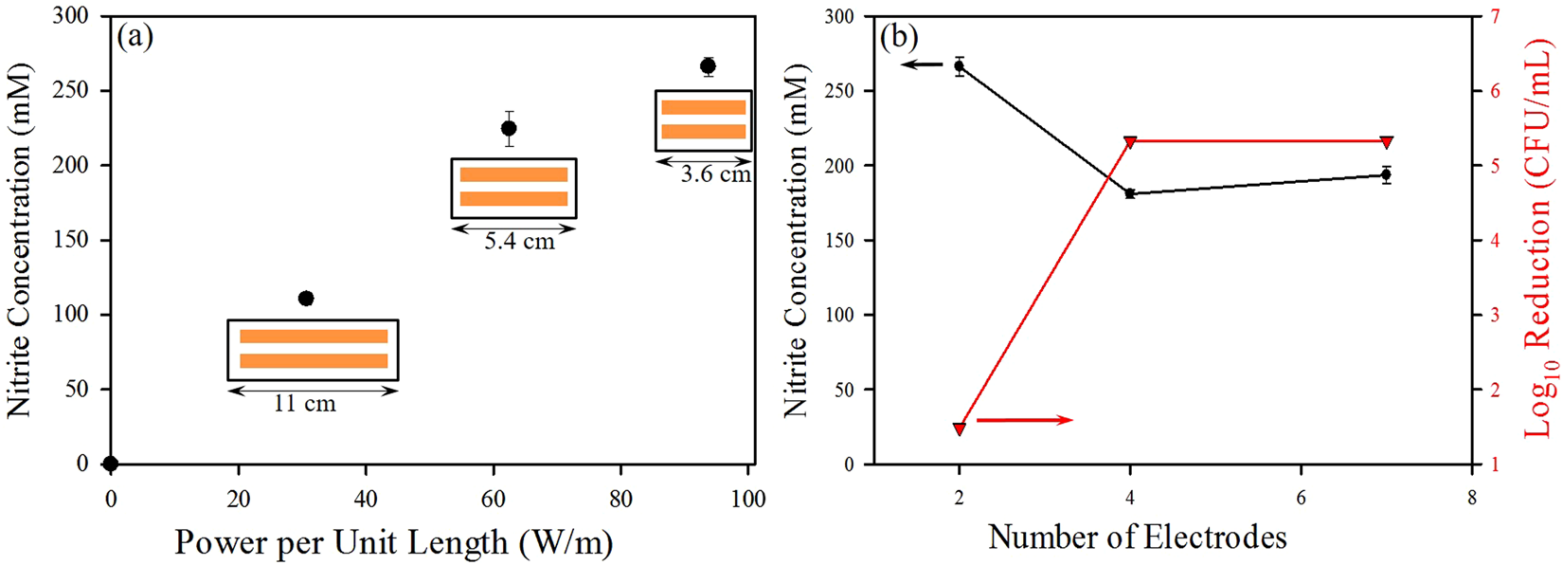
(**a**) Correlation of power density per unit length of electrode to nitrite production using a two electrode configuration by changing the electrode length; (**b**) Correlation of reduction in *Listeria monocytogenes* (5 strain mixture) with increase in number of electrodes for the same power density, with production of nitrites.

### Correlation of Nitrite Production and Bacterial Inactivation

The correlation of nitrite generation and bacterial inactivation was evaluated using a five strain mixture of *Listeria monocytogenes*. Additionally, the effect of increased plasma volume was investigated by varying the number of electrodes between 2, 4, and 7. Since the same net power was applied to all configurations, by increasing the number of electrodes, the volume of plasma produced increased while the power per unit length decreased. All tests were carried out after sufficient drying of bacterial suspensions on glass coverslips (approximately 60 minutes in a biosafety cabinet) so the observed results were on a relatively dry surface. However, the presence of moisture and humidity on the cellular level cannot be ruled out completely and further analysis is required to quantify the effect of moisture on bacterial inactivation with plasma treatment. A higher concentration of OH radicals were observed through optical emission spectroscopy in moist environments in a previous study^23^.

Nitrite concentration decreased with increasing electrode numbers from 2 to 4 and remained relatively the same between 4 and 7 (Fig. 2b). The 2-electrode configuration had a power per unit length of 93.75 W/m, as compared to the 15.63 W/m in the 7 electrode configuration. Therefore, an increased electrode number increased the plasma volume, albeit at lower power per unit length and therefore a reduced nitrite concentration.

Bacterial inactivation showed an opposite trend, increasing with an increase in electrode number at the same power input (decrease in power per unit length). Inactivation of the bacterial cells inoculated on glass coverslips was observed with the 4 and 7 electrode configurations after 2 minutes of treatment. The increased bacterial inactivation associated with increased plasma volume suggests no clear correlation between nitrite production and bacterial inactivation. A lower power per unit length still produced a high reduction of *L. monocytogenes*, in contrast to what was observed by others^40^. This finding suggests that SDBD device design, in which reactive species are actively pushed to distant surfaces by the induced flow, may be a contributing factor to bacterial inactivation^39,41–43^. The increase in decontamination effects with increased numbers of electrodes may be a consequence of overall increase in the relative densities of plasma-produced species other than RNA as a result of increased plasma volume. The results suggest that the low power, large plasma volume regime may be a better approach for sterilization and decontamination, thus making possible the development of low power plasma devices for decontamination applications.

### Correlation of Ion Density and Bacterial Inactivation

To investigate the role of ions in the plasma decontamination process, ion density was correlated with bacterial inactivation using a 3.6 cm, 4-electrode plasma actuator with treatment distances of 1, 3, 5, and 7 cm (Fig. 1). The 4-electrode configuration was selected since a substantially higher bacterial reduction was observed as compared to that measured with the 2-electrode configuration. Measurements from the air ion counter indicated that the ion densities at the treated surface were correlated with distances from the actuator, at approximately 2200 ions/cm^3^ at 1 cm and 400 ions/cm^3^ at 7 cm^44^. Ions are produced by the plasma process from secondary electrons near the actuator. These ions and electrons transfer energy to radicals and meta-stables, which are responsible for microbial inactivation and other cellular effects^24,32,40,41,45–48^. Sysolyatina *et al*. in their work noticed that electro physical effect of ions in corona discharges are not essential *per se* for bacterial inactivation but provides strong synergistic effects with other reactive agents (promoting their biochemical inactivation)^41^. They also reported that ROS were most efficient in bacterial inactivation^41^. Ozone, one of the primary meta-stable ROS produced, increased to more than 0.14 ppm within 10 seconds when plasma is generated by SDBD (data not shown).

**Figure 3:**
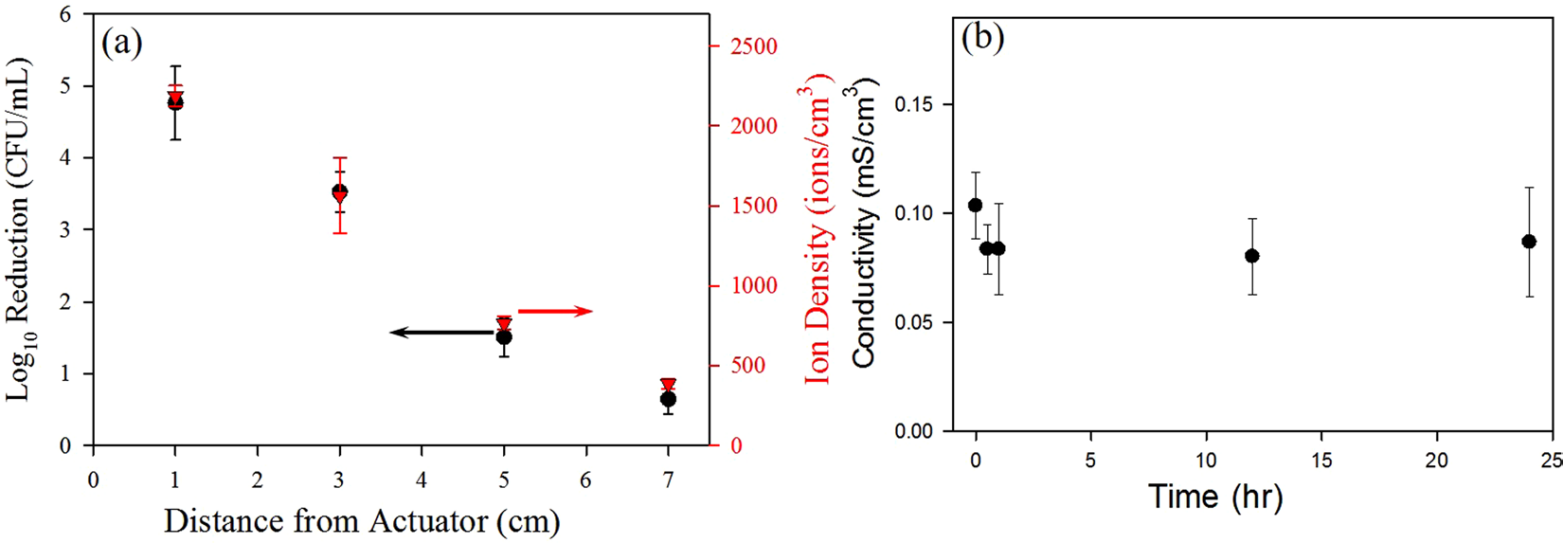
(**a**) Correlation of change in ion density and reduction in concentration of *Listeria monocytogenes* (5 strain mixture), with distance from plasma actuators using a four electrode configuration; (**b**) Change in conductivity of deionized water over 24 hours after a 4 minute treatment with plasma actuator using a two electrode configuration.

Bacterial inactivation decreased with decreased ion concentrations as the treated surface increased in distance from 1 to 7 cm from the actuators (Fig. 3a). Alternatively, bacterial inactivation increased with increasing electrode numbers for the same power input at the same distance although the magnitude of ions produced (~10^3^–10^4^) was the same for the 2, 4 and 7 electrode configurations at 1 cm. Hence, two conclusions can be drawn from these data: i) increased ion density correlates with increased bacterial inactivation; ii) the increased plasma volume produced by increasing electrode numbers from 2 to 7 does not increase ion concentration to a level that it affects bacterial inactivation. Rather, other plasma-produced reactive species must be contributing to the increased bacterial inactivation observed with increased plasma volume.

### Ion Accumulation in Aqueous Media

In addition to actuator distance, duration of exposure to ions is also a factor in plasma effects on cells, particularly in aqueous environments. To understand this factor, the lifespan of ions was measured in deionized (DI) water and untreated medium by observing the change in conductivity over time after different durations of plasma exposure (Fig. 3b). The conductivity in the untreated medium was significantly higher than that in DI water, which may be attributed to the presence of electrolytes and proteins in the medium. Hence, conductivity changes in the medium could not be measured. The ionic perturbations observed in DI water after plasma treatments were negligible. Untreated DI water retained a conductivity of 0 mS/cm^3^, even after 24 hours. The absence of change in the perturbations introduced in the conductivity of DI water post treatment showed that the conductivity created is a consequence of oxygen and nitrogen species rather than ions, which have a life span on the order of nanoseconds to milliseconds^41,49^. Therefore, the effects of ions in aqueous environments may be discounted as playing an important role.

Another possible explanation for these results is the generation of acidic H_3_O^+^ ions by reactions of the water molecules with H_2_O_2_^50^. Other researchers have noted an increase in acidity and formation of nitrous (HNO_2_) and nitric (HNO_3_) acid, along with H_2_O_2,_ in unbuffered water^43,51^. A similar decrease in pH was observed in previous work^23^ and was attributed to formation of HNO_2_ and HNO_3_, along with carbonic acid (H_2_CO_3_). Increased conductivity may be a result of dissociation of these acids since acidified aqueous nitrate and nitrite anions have been shown to form when water is exposed to atmospheric plasmas^43,52^.

No substantial increase in ionic perturbations (conductivity) with increased electrode numbers was observed, further suggesting that ions may not contribute to the cellular effects of plasma treatment but rather to the formation of nitrites and increase in the oxidative species, also contributing to increased acidity. The pH decreased from 7 to 3.6, 3.6, and 3.4 and remained constant over a period of 24 hours when exposed to plasma with the 2, 4, and 7 electrode configurations for 2 minute treatments at 1 cm, respectively. These results corroborate the theory of Kono *et al*. that a synergistic antimicrobial effect occurs as a result of NO_2_^−^, H_2_O_2_, and low pH^52,53^. ROS can also interact with NO to produce other reactive species that may contribute to the reduced pH, and accumulation of acids such as nitric acid^23^. In acidic environments, NO_2_^−^ and O_2_^−^ can also react to form peroxynitrous acid (ONOOH) and nitrous acid (HNO_2_)^54^. This is another example of how device design affects device performance, evident from the difference in trends of pH change observed here and that observed by Kojtari *et al*.^55^. A time course analysis is required for each of the electrode arrangements to ascertain the full effect on change in pH. These results suggest that in an aqueous medium the plasma-produced ions do not contribute to observed cellular effects.

### Extracellular Nitrite Accumulation

**Figure 4:**
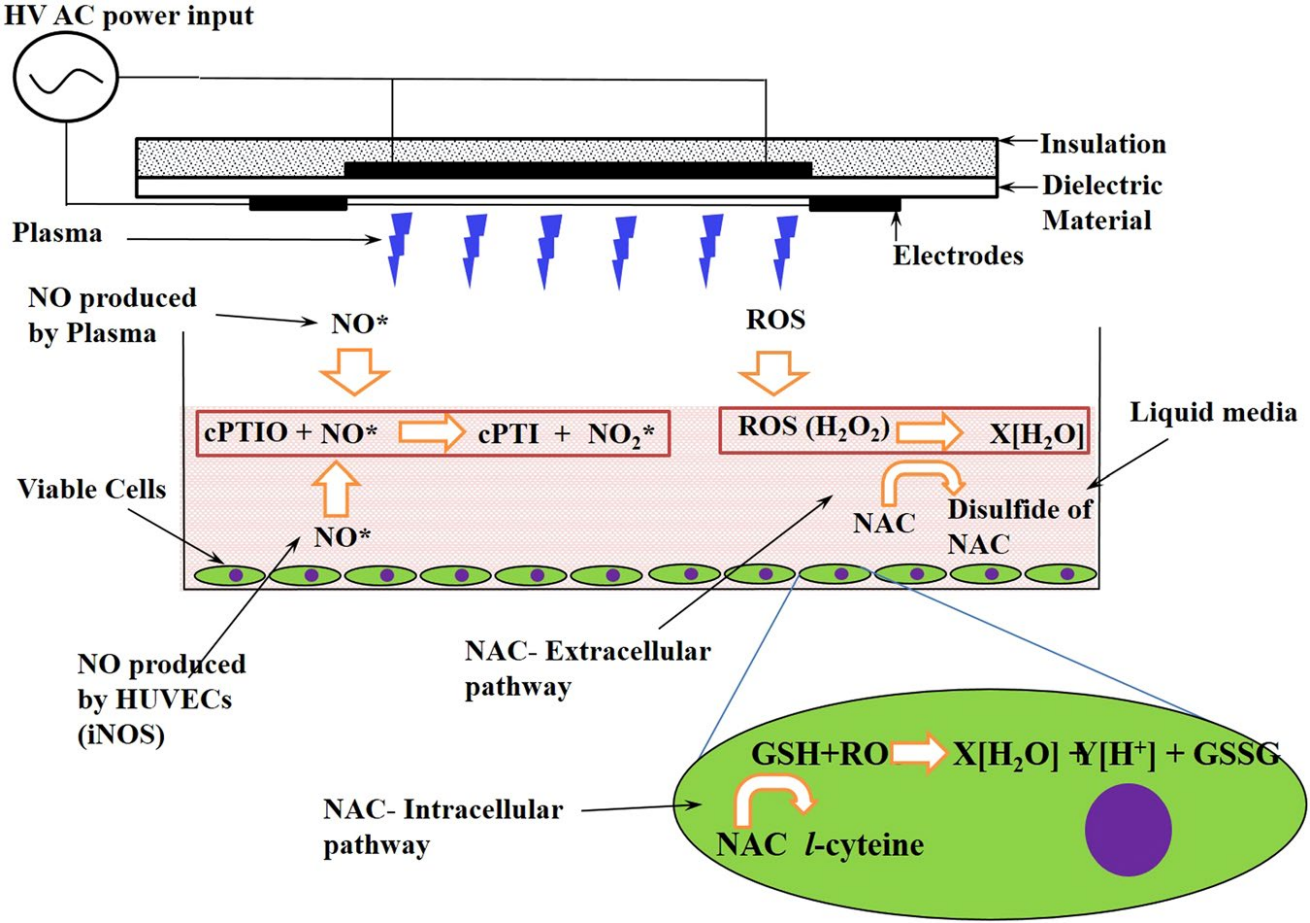
Schematic showing the cytoprotective interactions of ROS scavenger NAC (5 mM) with different ROS species (both intracellular and extracellular mechanisms) and NO with NO scavenger cPTIO (100 µM), respectively.

**Figure 5:**
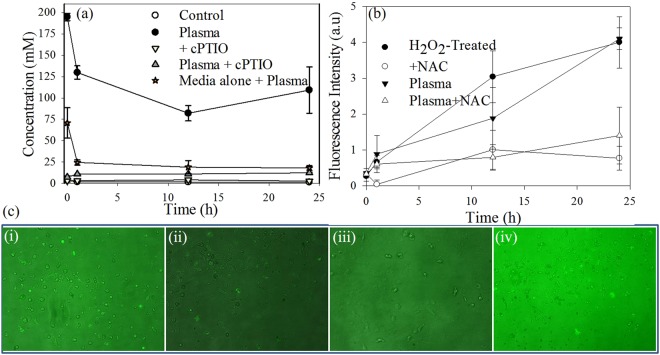
(**a**) Analysis of plasma induced nitrite concentration as a indicator for NO generation, with and without NO scavenger cPTIO; (**b**)Analysis of plasma induced intracellular ROS (Oxidative stress response) as a indicator for ROS generation, with and without ROS scavenger NAC (5 mM); (**c**) Fluorescent micrographs of HUVECs representing oxidative stress response to plasma with and without ROS scavenger NAC (5 mM). (**a**) Plasma treated HUVECs; (**b**) Plasma treated HUVECs with NAC (5 mM); (**c**) Positive control (200 µM H_2_O_2_); (**d**) untreated control with NAC (5 mM).

To evaluate the cellular effects of RNS, HUVECs in media were treated with SDBD cold plasma with and without the presence of a water-soluble NO scavenger, cPTIO. The resulting concentration of nitrites in the most stable configuration in aqueous media, the metabolite form of NO, was then measured via the Griess assay^38,56,57^ (Fig. 4). One hundred µM of c-PTIO was used since this concentration was effective at mitigating the effects of both extracellular and intracellular NO with no observable effects on the cells themselves^11,58^. In plasma, NO_2_ produced from NO and ozone (O_3_) reacts with H_2_O to form NO_2_^−^ and NO_3_^−^ ^59^. Nitrite concentrations in plasma treated samples containing HUVECs without the c-PTIO scavenger were nearly 10 fold higher than in samples containing both HUVECs and the scavenger (Fig. 5a), indicating that increased nitrite concentration is due to NO conversion to nitrite. Thirty minutes after plasma exposure of samples without the scavenger, nitrite concentrations were observed to be as high as 195 µM, decreased to approximately 130 µM after one hour, and stabilized to approximately 105 µM after 24 hours. HUVEC cell-containing control samples (no plasma exposure) with and without the scavenger had negligible nitrite concentrations for all time points. These results confirm that the increased nitrite concentration was not produced solely by the enzyme nitric oxide synthase (NOS) in HUVECs, but rather was a result of the plasma treatment. This finding corroborates those of another study in which high HUVEC cell viability was measured after a 4 min plasma treatment^23^.

**Figure 6:**
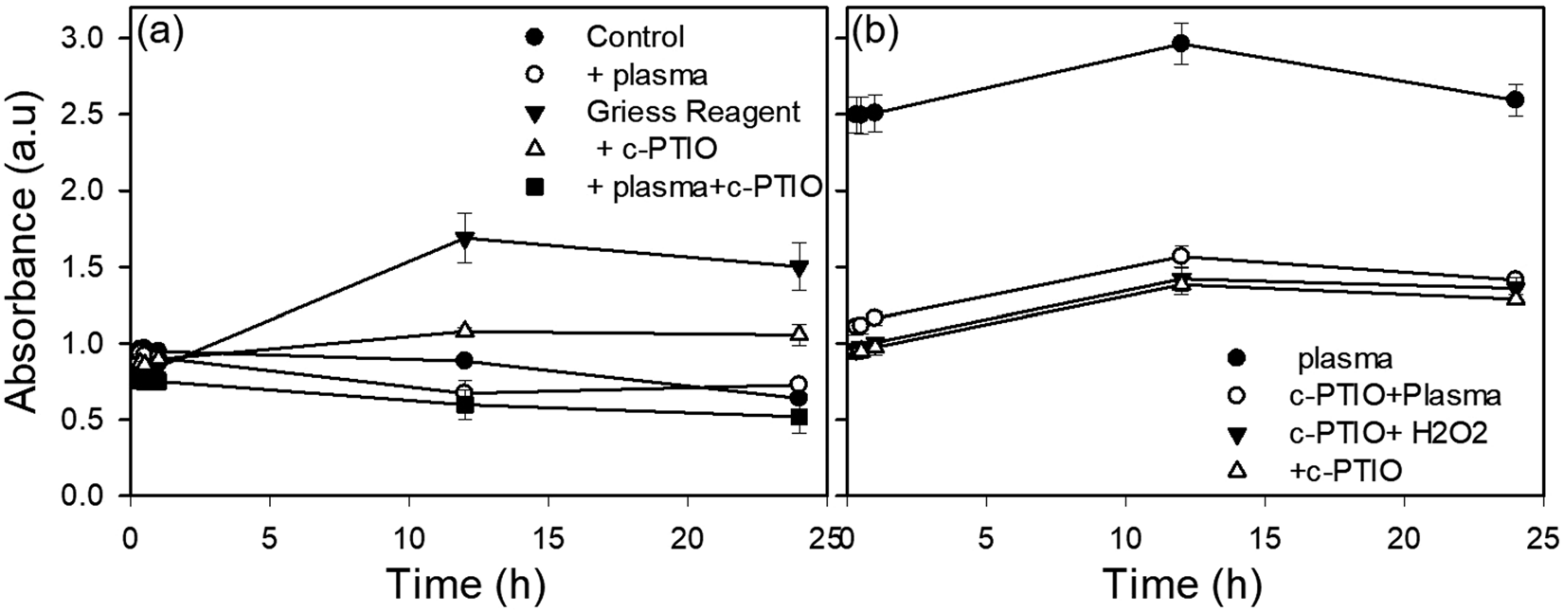
Analysis of plasma induced nitrite concentration as a indicator for NO generation in *P. aeruginosa*, with and without NO scavenger cPTIO; (**a**) Without Griess reagent (**b**) with Griess reagent.

When evaluating nitrite concentrations in HUVEC medium devoid of cells, a similar trend was observed. Nitrite concentrations in HUVEC medium without the scavenger were highest 30 minutes after plasma exposure, with an average of 70 µM, and then decreased to18 µM over a period of 24 hours. Therefore, since essentially the same increase in nitrite concentration immediately following plasma treatment and then a rapid decrease that leveled out after 1 hour was observed in HUVEC media with and without cells, it was concluded that NO produced by the plasma did not elicit a noticeable response from the HUVECs. The increased nitrite concentration of the media with the HUVECs may have been due to the presence of the cells themselves and a higher nitrate concentration baseline when compared to the nitrite concentration in the media alone. Similar results were observed with prokaryotic *P. aeruginosa* as well (Fig. 6).

### Intracellular Oxidative Stress Response

The cellular oxidative stress effects of ROS produced by SDBD plasma were evaluated using HUVECs in media. By adding a ROS scavenger (NAC) and a ROS indicator (carboxy-H_2_DCF-DA) to the media, the relative fluorescence intensity can be correlated with oxidative stress (Fig. 4). The extent of oxidative stress experienced by a cell depends on the concentration of ROS within a cell and the rate at which the ROS can be reduced. O_2_^−^ and H_2_O_2_ are produced during normal cellular respiration but are rapidly and efficiently reduced by several enzymes^60^. Oxidative stress occurs when these enzymes are not able to reduce ROS rapidly enough, causing an increase in ROS concentration and the potential for oxidative damage to DNA, lipids, and proteins. Although O_2_^−^ and H_2_O_2_ have the potential to cause oxidative damage themselves, they also serve as precursors to the more potent hydroxyl radical (OH^*^) through pathways such as the Fenton reaction^60^. OH^*^ is the most reactive of the ROS and rapidly causes oxidative damage to cellular components.

**Figure 7:**
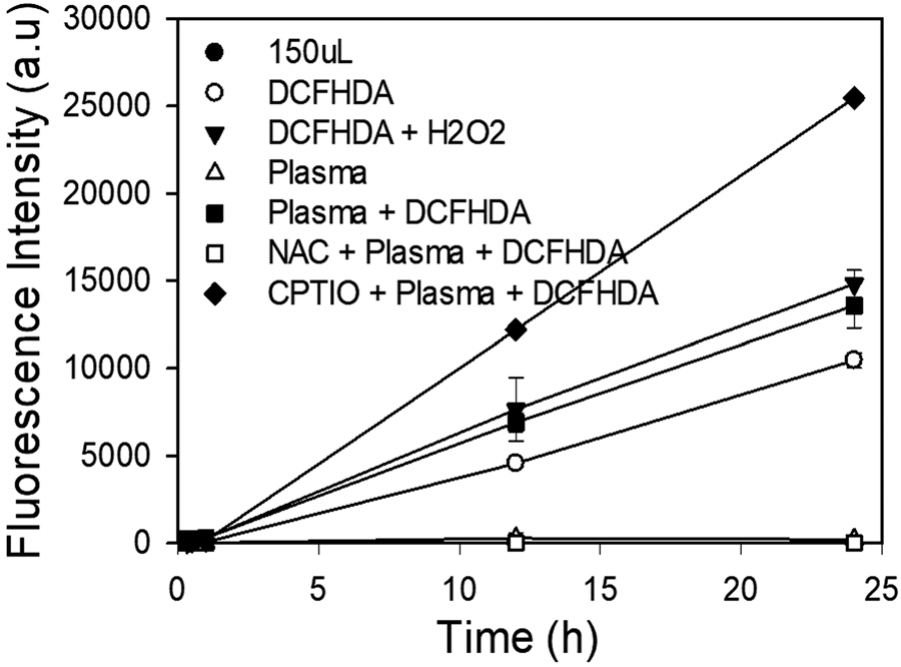
Analysis of plasma induced intracellular ROS (Oxidative stress response) in *P. aeruginosa* as an indicator for ROS generation, with and without ROS scavenger NAC (5 mM)

The effect of ROS generated by plasma treatment on HUVECs was observed by using carboxy-H_2_DCF-DA, with and without the presence of NAC (Fig. 5b). Positive control samples without NAC, containing 200 µM H_2_O_2_ (Fig. 5c(c)), had a fluorescence intensity similar to that of the plasma treated samples without NAC (Fig. 5c(a)). Fluorescent micrographs showed the highest fluorescence intensity for the plasma treated samples without NAC (Fig. 5c(a)) and the lowest for the untreated control samples with NAC (Fig. 5c(d)). Plasma treated samples with NAC (Fig. 5C(b)) showed a lower intensity than those without, indicating that NAC was able to neutralize the plasma generated ROS and reduce the level of oxidative stress experienced by the cells. Additionally, fluorescence intensity increased over time after plasma treatment and was highest after 24 hours, indicating the occurrence of oxidative stress within the cells. No significant ROS related stress was observed in any of the untreated control samples, with or without NAC. Similar results were observed with prokaryotic *P. aeruginosa* as well (Fig. 7).

Taken together, these results suggest two major conclusions. First, production of a relatively high concentration of ROS by SDBD plasma was confirmed, with the relative concentration (and therefore cellular effects) negatively correlated with increased distance from the actuators. Second, ROS produced by SDBD plasma was found to cause oxidative stress in HUVECs that continued to increase after plasma treatment was stopped.

Extracellular ROS generation by SDBD plasma actuators was assessed using optical emission spectroscopy in a previous work, wherein the major oxidative species were found to be O_3_, OH*, NO, and O_2_^+^ ^23^. These ROS can serve as precursors to other ROS, such as H_2_O_2_ and HNO_2_, in an aqueous medium^61^. Several authors have attempted to validate the dominance of HNO_3_ and H_2_O_2_ in the plasma interaction with cells, especially in the generation of plasma activated water (PAW)^51^. However, the general conclusion was that these agents, alone^62^ or in combination, did not give the same response as observed with plasma treatment^43,55,63^, thus indicating a multicomponent chemical dynamic.

### ROS and Plasma Effects on Bovine Muscle Tissue

The dominance of the role of ROS rather than RNS in the plasma treatment process was further confirmed by assessing the effects of plasm treatment on pathogen-inoculated bovine muscle tissue. Interestingly, negligible bacterial inactivation was observed on muscle tissue samples surface inoculated with *L. monocytogenes*. This observation may be partly attributed to the surface roughness and porosity of the muscle tissue, providing more shelter for the bacteria, or a shadowing effect, from the plasma generated species^64^. Alternatively, this observation may be a result of the high concentration of myoglobin and other components having high affinities for ROS. Pavlovich *et al*. observed similar results using pig skin, in which a slight reduction in *E. coli* concentration was observed after plasma treatment as compared to other substrates^39^. The presence of such compounds may reduce the concentration of ROS able to interact with and damage bacterial cells.

**Figure 8:**
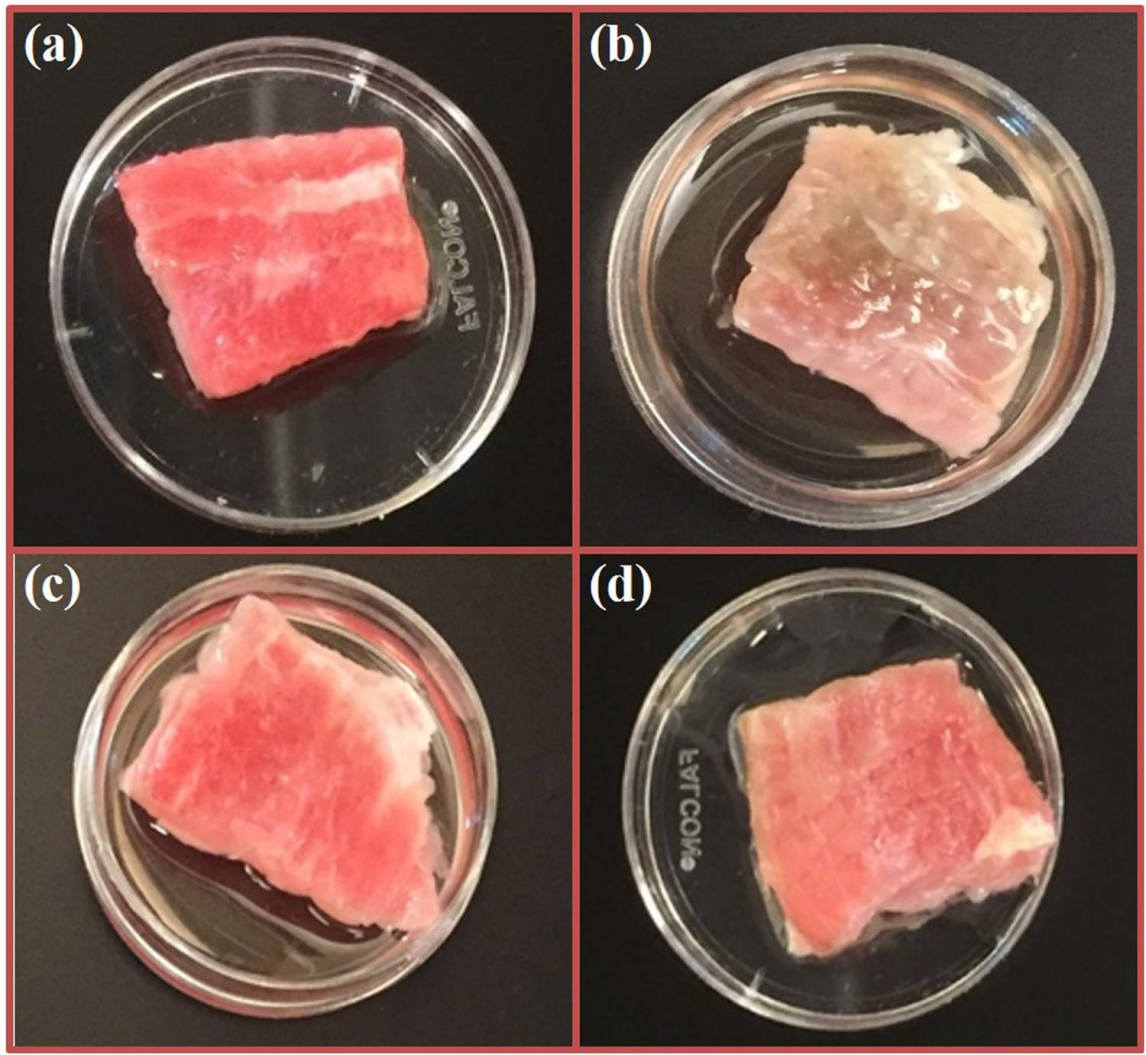
Effects of plasma exposure on bovine muscle tissue; (**a**) control (no NAC); (**b**) plasma treated (no NAC); (**c**) control (with 5 mM NAC); (**d**) plasma treated sample (with 5 mM NAC).

Although limited bacterial inactivation was observed, the texture and color of the muscle tissue noticeably changed from smooth to wrinkled and from bright red to a rustic brown. This texture and color change may be characteristic evidence of oxymyoglobin (OxyMb) oxidation to form methemoglobin (MetHb) by ROS^36^. The high concentration of iron in oxymyoglobin (or oxyhemoglobin) may contribute to the high affinity of ROS to oxymyoglobin^65^. Tang *et al*.^66^ reported a similar observation in which the addition of glutathione to bovine muscle cytosol improved oxymyoglobin redox stability. Further investigation of this observation was carried out by treating the muscle tissue samples with and without NAC, an ROS scavenger, prior to plasma treatment. Plasma treated samples with NAC showed no observable color or texture changes, whereas those without NAC exhibited the characteristic color change from bright red to rustic brown (Fig. 8). NAC produced no visibly detrimental effects on untreated muscle tissue control samples (Fig. 8(a)). These results confirm that SDBD produces a relatively high concentration of ROS, even beyond the plasma region itself, and is a major contributing factor to the cellular effects of plasma treatment. Additionally, the high affinity of myoglobin to ROS may have scavenged the plasma-produced ROS before they were able to have detrimental effects on *Listeria* cells on the surface of the meat, confirming the major role of ROS on bacterial inactivation. Further histological analysis of the tissue samples is required to better characterize the observed effects of cells subjected to plasma treatment.

### Cell Surface Effects of SDBD Treatment

**Figure 9:**
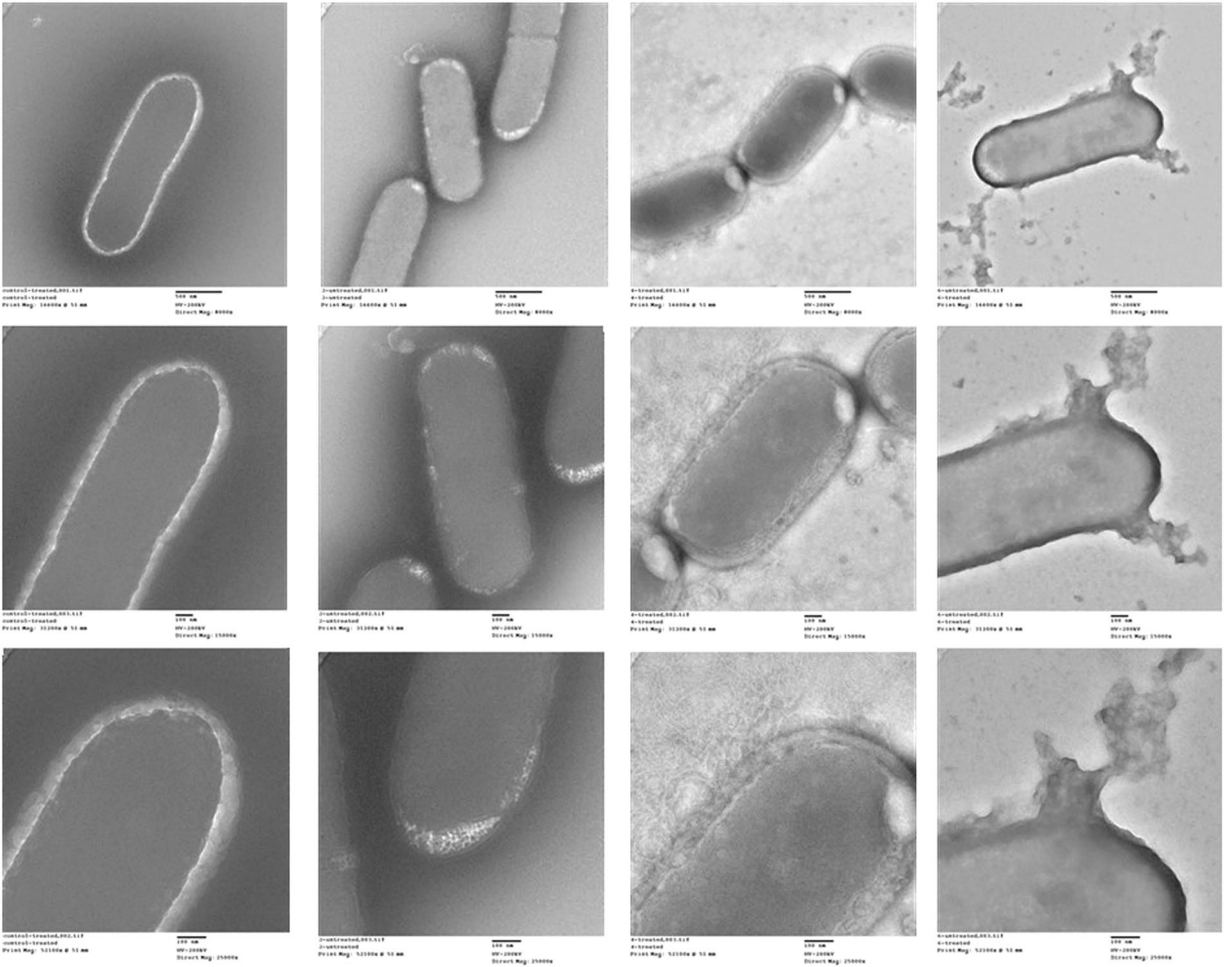
TEM of *Listeria innocua* treated with SDBD cold plasma for 2 and 4 min, compared with an untreated control.

TEM of SDBD plasma-treated *Listeria* cells compared to untreated controls revealed noticeable morphological differences with increasing treatment times ranging from 2 to 6 min (Fig. 9). Untreated cells had distinct boundaries when clustered in groups, with multiple cells undergoing mitosis and clearly visible fimbriae under high magnification. After 2 min treatments cell surfaces were visually darker and cell boundaries appeared more ragged, less uniform, and less distinct between individual cells. Substantially more extracellular debris, visible only in treated samples, may be a result of membrane damage and cytosol leakage. Membrane and cell surface damage became increasingly more evident after 4 and 6 min treatments, evidenced by darker staining, less distinct cellular margins, and greater amounts of extracellular debris (Fig. 9).

### Summary and Conclusions

A differential response to plasma treatment between prokaryotic and eukaryotic cells has been observed by several researchers^6,23,31^, Compared to eukaryotes, prokaryotic cells are much more sensitive to oxidative stress^67^. A similar differential response has been observed between normally functioning eukaryotic cells and cancer cells^68^, attributed to the Warburg effect, by which cancer cells are damaged more readily by ROS due to their increased reliance on aerobic metabolism^69,70^. Based on this characteristic, a selective treatment of cancer cells among healthy mammalian cells may be possible^68,71^. The rate of cellular inactivation by plasma generated in air or oxygen was several times faster than when it is generated in noble gases, further corroborating the dominant role of ROS on cellular effects^72^. Identifying and quantifying the specific ROS produced will aid in further tuning of the system for higher precision and effective applications. Since non-thermal plasmas allow a surface treatment effect rather than a bulk effect, it will be beneficial to tune the system for etching-like capabilities, providing an exact treatment approach. Furthermore, an increased understanding of the utilization of the flow induction capabilities of SDBD for long range applications should be investigated further and be more fully characterized.

To summarize, these results provide evidence that ROS, rather than NO and ions, are the major contributors to SDBD plasma-induced bacterial inactivation and eukaryotic oxidative stress. Maximal cellular effects were observed when samples were placed in close proximity to plasma actuators, possibly the result of a synergistic effect of ROS, NO, and ions that decreased with increasing distance from the actuators, as was also observed by Kono *et al*.^53^. The observed ion density was highest immediately adjacent to the plasma region and decreased rapidly with increased distance from the actuators. In aqueous medium, ROS, NO, and ions contributed to a decrease in pH, adding to the synergistic effects. At increased distances from plasma actuators, ROS were the major plasma generated species interacting with treated cells.

Nitrites may be beneficial in higher concentrations for wound care applications such as in acidified nitrite creams for topical NO donating wound healing agents^73^ and surface disinfection of robust, difficult to inactivate bacterial strains^74^. Direct plasma exposure methods such as volumetric DBD, although sometimes more efficient at bacterial inactivation, have been observed to cause tissue damage and often provides a non-uniform treatment^39^. Hence, the semi-direct method of plasma exposure used in this work (SDBD) is a good alternative with capabilities of flow control to push the generated plasma species to the surface being treated. SDBD actuator designs also allow manipulation of the species being generated by changing the plasma parameters and electrode configurations, thus providing a specific desired effect (i.e. sterilization vs. wound healing). Similar studies, such as the one in the work of Lunov *et al*.^75^, provide insight into what effects different kind of changes in various parameters of a plasma generation method can produce. The selectivity and tuning capabilities offered by this technology can help in our efforts to resolve major global public health issues such as the development of bacterial antimicrobial resistance, chronic wound infections, and sterilization of a variety of both organic and inorganic surfaces. Optimized SDBD actuators are viable candidate for numerous applications in the healthcare industry, especially for sterilization and wound healing.

## Materials and Methods

### Plasma Actuator Arrangements

All SDBD plasma actuators were constructed as described previously^23^. This plasma actuator arrangement has been shown to produce reactive species and UV light. However, previous findings using optical emission spectroscopy has shown UV light production to be negligible^23^ so it was not evaluated in this study. Briefly, SDBD plasma actuators were constructed using 0.0254 cm thick Teflon sheets (McMaster-Carr Supply Company, USA) as a dielectric medium, to which 0.2 cm wide copper tape (McMaster-Carr Supply Company, USA) was attached asymmetrically on either side to serve as electrodes (Fig. 1). All experiments were carried out with actuators operating at 6.75 W using a high voltage, high frequency transformer (Minimax70, Information Unlimited, Amhert, NH). Different configurations of electrodes were used, as described below.

(i) To test the effects of power density on nitrite production, three different actuators were constructed with two powered electrodes on one side of the dielectric with lengths of 3.6 cm, 5.4 cm, and 11 cm. Power per unit length of the electrode was calculated for each configuration using the equation:

$$Power\,per\,unit\,length(\frac{W}{m})=\frac{Input\,Power\,(W)}{Total\,length\,of\,plasma\,(m)\,}$$

where the total length of plasma is measured by multiplying the number of edges on which the plasma is generated by the length of the electrodes. Accordingly, power densities per unit length of 93.75 W/m, 62.5 W/m and 30.68 W/m were observed for electrode lengths of 3.6 cm, 5.4 cm, and 11 cm, respectively.

(ii) To understand the bactericidal effects of increased electrode numbers at a constant electrode length, three actuators were prepared using 3.6 cm long electrodes on a dielectric of area 5 cm × 4 cm. The numbers of electrodes placed on the dielectric were 2, 4, and 7, with the same power input as stated above. Samples were exposed to plasma at 1 cm from the actuator for 2 min. To evaluate the effect of increased distance between the actuator and the sample, the 4-electrode configuration was used at distances of 1, 3, 5, and 7 cm from the sample.

(iii) For mammalian cell culture experiments, the actuator size had to be adjusted to fit the culture plates, as in a previous study^23^. In brief, two parallel electrodes of 11 cm × 0.5 cm were placed on 12.5 cm × 3 cm Teflon sheets on the side exposed to the sample being treated. A common ground electrode of 11 cm × 0.5 cm was used on the opposing side, placed asymmetrically in relation to the powered electrodes (Fig. 1). The actuator was placed 1 cm from the surface of the cell monolayer formed on the bottom of the petri dish. In accordance with previous work^23^, all HUVECs were treated with plasma for 4 min.

### Air Ion Production by SDBD Actuators

The density of ions generated due to SDBD cold plasma actuators were measured with an air ion counter (AlphaLab, Inc., model AIC, Salt Lake City, UT), which measures separately the number of positive and negative ions per cm^3^ of air. This air ion meter is based on a Gerdien Tube (Gerdien Condenser) design and contains a fan that pulls air through the meter at a calibrated rate. The density of ions resulting from plasma exposure with a 4-electrode actuator was measured at 1, 3, 5, and 7 cm from the actuator surface. The air ion density resulting from plasma generation was compared to the ambient air to determine the relative increase in ion density. All measurements were done in triplicate and with the fan off to prevent any bias due to induced convection. Ion density was measured once a steady state reading was observed by the air ion counter.

### Bacterial Inoculation and Treatment

Five strains of *Listeria monocytogenes* (F6854, 12433, G3982, J0161 and, Scott A) were cultured for 24 hours at 37 °C in tryptic soy broth (TSB, Difco). After incubation, 1 mL of each culture was centrifuged at 9,000 x g for 3 minutes. Pellets were re-suspended in 1 mL of 0.1% (w/v) sterile peptone water (Difco) and combined to obtain a 5-strain mixture (5 mL total). Multiple-strain mixtures were used in this research to more closely imitate real-world populations of bacteria consisting of more than a single strain and to rule out any strain-specific responses to the plasma treatment. The mixture was then diluted 10-fold to produce the desired inoculum concentration of 10^7^ CFU/mL, and 100 µL was uniformly distributed in 20–25 spots on sterile 2.2 cm^2^ glass coverslips (10^5^ to 10^6^ CFU/spot). Inoculated coverslips were air dried in a biosafety cabinet for approximately 60 min prior to plasma treatment. Dried bacterial cells were evaluated in this study to mitigate the effects of medium acidification when bacterial cells are treated in a suspension and allow the more short-lived ROS and RNS to interact with the cell surface rather than the medium in which the cells are suspended. Sysolyatina *et al*. reported composition of these secondary species is not the same as compared with primary gaseous species generated by the plasma source^41^, hence drying the bacteria on coverslips helped determine the effects of the primary species. The 100 µL inoculum was spread out in 20–25 spots to reduce the accumulation of layers of cells on one side of each spot as the liquid evaporates as a result of surface tension.

Inoculated coverslips were treated in triplicate for two separate experiments: (1) 1, 3, 5, and 7 cm with 4-electrode actuators for 2 min and; (2) 2, 4, and 7-electrode actuators at 1 cm for 2 min. Plasma-treated and untreated control inoculated coverslips were washed by vortexing for 30 s in 10 mL 0.1% (w/v) sterile peptone in 50 mL conical tubes. Wash fluids were 10-fold serially diluted in 0.1% peptone for enumeration, for which 100 µL of each dilution was plated in duplicate on TSA and incubated overnight at 37 °C. Bacterial inactivation due to cold plasma treatment was assessed by comparing the bacterial recovery of plasma-treated samples to untreated controls. This method was used to negate the potentially negative effects of desiccation on cell viability.

For extracellular nitrite detection and intracellular oxidative stress detection in prokaryotic cells, cryopreserved clinical isolate of Pseudomonas aeruginosa obtained from canine ear swabs were used for the study. *P. aeruginosa* was revived on blood agar plates. Following revival, roughly 10 colonies were picked a nd mixed in 2 ml of 1x PBS. A serial dilution was made to estimate the concentration of the mixture. Under a dark environment, 50 mM DCFDA, 100 µM c-PTIO, 5 mM NAC were made. 2 mLof all these solutions were aliquot into different tubes. Roughly 10 colonies were added to all these tubes. In a 96 well plate, these reagents were added sequentially in triplicates. Negative and positive controls were established as per need.

All the readings were measured at 0 minutes, 10 minutes, 30 minutes, 60 minutes, 12 hours and 24 hours after final incubation. The concentration of *P. aeruginosa* used with each reagent was estimated using the Miles-Misra technique. The concentration was found to be 3–5 × 10^9^/mL.

### Human Umbilical Vein Endothelial Cell Culture

HUVEC were chosen for this study since they have been previously noted to be resilient to SDBD plasma treatment and show a dose-dependent response with higher viability compared to other common eukaryotic cells^23^. HUVEC derived from single donors (Life Technologies) were cultured in Medium 200 phenol red free (PRF), supplemented with low serum growth supplement (LSGS, containing 2% v/v fetal bovine serum, 1 µg/mL hydrocortisone, 10 ng/mL human epidermal growth factor, 3 ng/mL basic fibroblast growth factor, and 10 µg/mL heparin) following the vendor’s instructions, as described previously^23^. HUVECs were maintained at 37 °C, 5% CO_2_/95% air, in a humidified cell culture incubator and fed with fresh medium every 36 hours. When confluent, cells were suspended with 0.025% trypsin and 0.01% EDTA in PBS and neutralized with trypsin neutralizer solution (phosphate-buffered saline (PBS) containing calf serum as a trypsin inhibitor), centrifuged at 125 × g for 5 minutes, and re-suspended in growth medium. Viable cells were counted using trypan blue stained cells in a hemocytometer and seeded into various culture plates as required. Based on viability analysis and the minimal observed pH change of the HUVEC medium^23^, cells were exposed to plasma for 4 minutes at a distance of approximately 1 cm.

### Electrical Conductivity of Plasma Treated Water and Media

Ten mL of water or growth medium used for HUVEC culture were treated with plasma for 2 and 4 minutes. The electrical conductivity of the treated water and growth medium was measured with a portable pH/EC/TDS meter (Milwaukee Instruments, Inc., model MW802, Rocky Mt, NC) at 30 min, 1 hr, 12 hr, and 24 hr after plasma treatment.

### Extracellular Nitrite Detection in HUVEC

HUVECs were seeded into a 24 well plate at 12,000 cells per well and incubated with 500 µL medium. After 24 hours, cells were pretreated with carboxy-PTIO (100 µM), which scavenges NO stoichiometrically, and incubated for 45 minutes before they were exposed to plasma. Untreated samples with and without the NO scavenger were used as controls. Culture medium was retrieved at 30 min, 1 hour, 12 hours, and 24 hours for nitrite analysis and mixed with an equal volume of Griess Reagent (for nitrite detection) as specified by the vendor (Molecular Probes, Life Technologies, USA), then incubated at room temperature for 20 min. The absorbance was measured at 490 nm with an Emax precision microplate reader using the software SoftMax Pro 4.3 (Molecular Devices, Sunnyvale, CA), using a calibration curve with a range of 0–100 µM concentrations of NaNO_2_.

Fresh medium was used to measure nitrite production induced by plasma to establish a baseline nitrite concentration attributable to plasma exposure to discount nitric oxide synthase (NOS) activity. For measuring the effects of power density and number of electrodes, tests were conducted using 1 mL of medium in a 6-well plate and nitrite concentrations were measured 1 hour after exposure.

### Extracellular Nitrite Detection in Bacterial Cells

*P. aeruginosa* incubated with c-PTIO for one hour was seeded to a 96 will plate in triplicates. They were then incubated at 37 °C for 5–10 minutes. Equal volume of Griess reagent obtained commercially, was added to this mixture. Following a 5-minute incubation after adding Griess reagent, the plates were measured at 490 nm using SpectraMax microplate reader and SoftMax pro 4.6.

### Intracellular Oxidative Stress Detection in HUVEC

The intracellular effects of ROS produced by SDBD plasma were evaluated using HUVECs in media, but with the addition of a ROS scavenger (NAC) and a ROS indicator (carboxy-H_2_DCF-DA) (Fig. 4). Carboxy-H_2_DCF-DA is an acetylated form of fluorescin that is deacytelated by ROS, causing it to fluoresce. Thus, relative fluorescence intensity can be correlated to the relative intracellular accumulation of ROS. At the intracellular level, NAC is rapidly hydrolyzed to L-cysteine, allowing increased production of glutathione (GSH), a powerful antioxidant. In the presence of glutathione peroxidase (GSH-Px), GSH and H_2_O_2_ react to form disulfide (GS-SG) and water^76^ **(**Fig. 4**)**. At the extracellular level, NAC simply acts as a nucleophile, donating an electron to ROS introduced by the plasma treatment^76^. Five mM of NAC was used in this study since this concentration was found to provide effective cyto-protective properties while also not having toxic effects on the cells^58,77^.

HUVECs were seeded in four 96 well plates at a density of 7,000 cells per well and incubated overnight with 200 µL medium. NAC readily binds with all ROS, including those that contain nitrogen^78^, both intracellularly and extracellularly^76^. Four different conditions were created: (1) untreated negative control with NAC (5 mM^58^); (2) plasma treated cells without NAC; (3) plasma treated cells with NAC; (4) untreated positive control with H_2_O_2_ (200 µM). Intracellular ROS levels were assessed using the Image-IT LIVE Green Reactive Oxygen Species Detection Kit (Molecular Probes, Life Technologies, USA) according to the manufacturer’s protocol. In brief, Caboxy-H_2_DCF-DA was added to all cultures at 50 μM. Cells were incubated for 45 min, washed with PBS and 200 µL of media was added to each well. The media was removed at the time of exposure, with a thin layer to keep the cells hydrated as in the previous work^23,79^. For conditions containing NAC, media contained 500 mM of the scavenger. Fluorescence intensity was measured with a microplate reader, SpectraMAX GEMINI XS at 495/529 nm, using the software SoftMax Pro 4.3 (Molecular Devices, Sunnyvale, CA). Fluorescence images were captured using an inverted microscope with fluorescent lamp (Nikon Eclipse TE 2000-U, Melville, NY). Intensity was measured and images taken at 30 min, 1 hour, 12 hours, and 24 hours after plasma treatment. Each plate was only exposed once to eliminate photobleaching.

### Intracellular Oxidative Stress Detection in Bacterial Cells

*P. aeruginosa* mixed in 2 ml of 5 mM NAC was added to 96 well plates in triplicates. These plates were then incubated for 15 minutes. Following incubation, under a dark biosafety cabinet hood, equal volume of DCFDA was added. The plates were then covered and incubated for 5–10 minutes. Fluorescence was measured at 480/520 nm using SpectraMax Microplate reader and software SoftMax pro 4.6.

### Evaluation of ROS and Plasma Effects on Bovine Muscle Tissue

Two separate sets of experiments were designed to evaluate the role of plasma generated ROS on muscle tissue. First, the tissue was inoculated with pathogens (5 strain cocktail of *Listeria monocytogenes*) and exposed to plasma for 4 minutes with a 4 electrode configuration at 1 cm from the sample. In the second experiment, the muscle tissue was coated with 1 ml of 5 mM NAC prepared in PBS to observe if the effects of plasma were due predominately to ROS. Samples that did not have NAC were coated with PBS alone to prevent desiccation. Treatment conditions were the same as those for the first experiment.

### Transmission Electron Microscopy

Transmission electron microscopy (TEM) was used to visualize untreated control and plasma-treated *Listeria innocua* (a non-pathogenic species with similar cell morphology to *Listeria monocytogenes*) cells to evaluate the morphological effects of the plasma treatment. A suspension of approximately 10^7^ CFU/mL of *Listeria* cells, prepared as described above, were spotted onto carbon-backed gold TEM grids placed onto sterile glass coverslips, air-dried for 60 minutes, and treated with SDBD plasma actuators for 2, 4, and 6 minutes at a distance of 1 cm, as described above. The cells on untreated control and treated TEM grids were then negative stained with phosphotungstic acid and visualized with a JEOL JEM-2100 scanning transmission electron microscope system.

### Statistical Analysis

All experiments were conducted in triplicate and reported values are represented as mean ± SD. Significant difference between two groups was analyzed using ANOVA with 95% confidence interval. Differences in the results were considered statistically significant when *P* < 0.05.
